# Episiotomy in Dystocia

**Published:** 1881-07-20

**Authors:** F. O. Nagle

**Affiliations:** Philadelphia


### A CASE OF DYSTOCIA—RELIEF BY EPISIOTOMY.

BY F. O. NAGLE, M. D., OF PHILADELPHIA.

Labor may be rendered difficult by many and various causes. They are generally arranged into two classes. Those which render labor difficult by causes referable to the mother, and those in which the child is at fault.

The following case of difficult labor is one which belongs partly to both classes; where the labor was made difficult, on the part of the child, by an unusually large, healthy head; and on the part of the mother, by a small vulva.

The patient, a young married lady, twenty-six years of age, primiparæ, had engaged, and was attended by Dr. Wm. M. Caterson. The Doctor was sent for about 10 p. m., Sunday, April 3d. The patient stated to him that the pains came on Saturday evening. They were sharp, cutting pains, referred to the uterus, coming and going every twenty minutes, keeping her awake all the night. Sunday she continued to have them from time to time, not as often as before. In the evening they again became worse, and when the doctor arrived she was having them about every five minutes.

The doctor remained all night with her, and part of next day, very little progress being made during that time, and not wishing to bear the entire responsibility, he desired a consultation with me, which was granted.

I was called about 5 p. m., on Monday. At my first visit I found the patient in bed. Upon questioning her I obtained the following information: This was her first pregnancy; thought she was two weeks before her time. She was in labor forty-eight hours. The pains during my stay were feeble and slow, but severe enough to keep her almost in continual suffering. In making an examination *per vaginam*,

I found the head presenting at superior strait, second position of vertex. Os dilated to about two inches in diameter. Membranes intact. Fæces in rectum could be felt through posterior wall of vagina. Abdomen considerably enlarged.

Patient was told there was no occasion, at present, for alarm. She was ordered fifteen grains of quinine as a uterine stimulant, and an enema of soap and water. I left, and promised to return later in the evening.

I saw her again at half-past eight p. m., when the doctor informed me that about one hour after taking the quinine the pains became more severe, lasting longer and more effective. The os was dilated to about three inches in diameter. Presenting part engaged in pelvis. Rectum empty.

At my next visit, 10:45 p. m., I was told that the membranes had just ruptured. Pains were of a bearing-down character. Os fully dilated. Large head diagnosed. Patient had vomited during my absence; she was ordered seven grains more of quinine. The head slowly descended to the perineum. From the rupture of membranes to the appearance of head at vulva four and three-fourths hours elapsed. During this time the pains were strong and severe. When the head was low down, and the perineum distended to its utmost, it was evident the head could never be born without a rupture, owing to the smallness of the orifice of vulva. I tried to save perineum by endeavoring to deliver head during the absence of pains, as taught by Prof. R. A. F. Penrose. The head could not be born, as the vulva was too small, rigid and inelastic.

The application of forceps was considered, but the danger of rupture by their use was evident.

I obtained consent of the patient to perform episiotomy. This I did with a pair of scissors, making a cut on each side of the vulva about one and a half inches from median line of perineum. This procedure removed the tension of vulva, and allowed the head to pass out readily, saving, at the same time, the perineum, the fourchette, even, not being torn. Passing my hand around the neck, I discovered the cord encircling it. This was easily slipped over head. The shoulders and body were delivered without any difficulty.

The child was an unusually large and well developed one; the head being particularly large. Smacking the child with the hand was all that was necessary to establish respiration.

The cord was cut, and placenta delivered by Crede's metnod. No stitches were applied to cuts, the wounds healing by granulations. There was no hemorrhage from the incisions nor from the uterus.

Child born 3½ a. m., Tuesday, April 5th.

*Duration of Labor.*—First stage. Dilatation of os, 53 hours. Second stage. Expulsion, 4¾ hours. Third stage. Delivery of placenta, 15 minutes. Total, 58 hours.

*Weight and Measurements of Child.*—Weight, 12½ pounds. Length, from vertex to buttocks, 15½ inches; from buttocks to soles of feet, 10 inches. Entire length, 25½ inches.

*Circumference of Head.*—Tape passed from occipital ridge above ears to frontal bones, 15¼ inches.

*Diameters of Head.*—Bi-parietal, 4 inches; occipito-mental, 6 inches; fronto-mental, 4 inches; occipito-frontal, 5 inches; bis acromion, 5 inches; bis iliac, 4 inches.

The cause of this lingering labor was, I believe, the excessive distention of the uterine cavity by the large fœtus Just the same as we have in dropsy of the amnios, where the uterine walls are over-distended, making them thinner and preventing them from acting with energy.

In primiparæ, delivering the head during the absence of pain will, in the majority of cases, save the perineum from rupture. Where this cannot be done, cutting the vulva, as in this case, is the only resort. The edges can be united by a stitch. None were used in this case. No deformity followed.

I would say, in conclusion, that the action of quinine in this case, as a uterine stimulant, was a very decided one. I have seen it used in similar cases, and always with a very happy effect. I consider it, in full doses (15 gr.), a very valuable aid in cases where the pains are insufficient. Its mode of action is not yet fully understood. It is believed, however, that it does not originate uterine contractions, merely stimulating those present.

The lying in of the patient was not attended with anything unusual. Mother and child are both well.—*Med. and Surg. Reporter.*

